# A customized fixation plate with novel structure designed by topological optimization for mandibular angle fracture based on finite element analysis

**DOI:** 10.1186/s12938-017-0422-z

**Published:** 2017-11-15

**Authors:** Yun-feng Liu, Ying-ying Fan, Xian-feng Jiang, Dale A. Baur

**Affiliations:** 1 Key Laboratory of E &M (Zhejiang University of Technology), Ministry of Education & Zhejiang Province, Hangzhou, 310014 Zhejiang China; 2 Department of Oral and Maxillofacial Surgery, School of Dental Medicine, Case Western Reserve University, Cleveland, OH 44106 USA

**Keywords:** Mandibular angle fracture, Customized plate, Topological optimization

## Abstract

**Background:**

The purpose of this study was to design a customized fixation plate for mandibular angle fracture using topological optimization based on the biomechanical properties of the two conventional fixation systems, and compare the results of stress, strain and displacement distributions calculated by finite element analysis (FEA).

**Methods:**

A three-dimensional (3D) virtual mandible was reconstructed from CT images with a mimic angle fracture and a 1 mm gap between two bone segments, and then a FEA model, including volume mesh with inhomogeneous bone material properties, three loading conditions and constraints (muscles and condyles), was created to design a customized plate using topological optimization method, then the shape of the plate was referenced from the stress concentrated area on an initial part created from thickened bone surface for optimal calculation, and then the plate was formulated as “V” pattern according to dimensions of standard mini-plate finally. To compare the biomechanical behavior of the “V” plate and other conventional mini-plates for angle fracture fixation, two conventional fixation systems were used: type A, one standard mini-plate, and type B, two standard mini-plates, and the stress, strain and displacement distributions within the three fixation systems were compared and discussed.

**Results:**

The stress, strain and displacement distributions to the angle fractured mandible with three different fixation modalities were collected, respectively, and the maximum stress for each model emerged at the mandibular ramus or screw holes. Under the same loading conditions, the maximum stress on the customized fixation system decreased 74.3, 75.6 and 70.6% compared to type A, and 34.9, 34.1, and 39.6% compared to type B. All maximum von Mises stresses of mandible were well below the allowable stress of human bone, as well as maximum principal strain. And the displacement diagram of bony segments indicated the effect of treatment with different fixation systems.

**Conclusions:**

The customized fixation system with topological optimized structure has good biomechanical behavior for mandibular angle fracture because the stress, strain and displacement within the plate could be reduced significantly comparing to conventional “one mini-plate” or “two mini-plates” systems. The design methodology for customized fixation system could be used for other fractures in mandible or other bones to acquire better mechanical behavior of the system and improve stable environment for bone healing. And together with SLM, the customized plate with optimal structure could be designed and fabricated rapidly to satisfy the urgent time requirements for treatment.

## Background

Mandibular angle is located at the junction of the ramus and the lower body of the mandible, and the bone here is very weak, so it is easily fractured from violent crimes, sport or traffic accidents, or pathological processes [1–3]. According to literature statistics, the mandibular angle is one of the most common sites for fractures, accounting for 23–42% of all cases of mandibular fractures [4]. Moreover, mandibular angle fractures have the highest postoperative complications among all mandibular fractures, and the loosening of screws and fracturing of plates are main reasons for the complications [5].

Currently, there are two typical treatment modalities for the mandibular angle fractures: the first one uses one mini-plate for fixation, which has been widely used during the past two decades, following the principles described in 1975 by Champy [6–8]; the other one uses two mini-plates [9], with a upper mini-plate fixed at the same place of previous treatment which corresponds to the tension band of the mandible, and the lower mini-plate fixed at the inferior border of the mandible which corresponds to the compression band of the mandible. However, all the mini-plates used in the clinical case are standard and straight plates, and need to be bent to bone surface before fixation, which will increase the time of the operation and may lead to mismatching between the bone surface and titanium plates [10]. Based on recent clinical statistical studies, the incidence of plate removal has risen to 18%, occurring typically in less than 6–9 months after surgery, so the stability provided by the mini-plates has become a hot issue among surgeons [4, 11].

A few researchers had designed some custom-made or three-dimensional (3D) mini-plates by changing the hole size, distance between the holes and shape of the plate for mandibular angle fractures [11–13]. However, these 3D plates are only modified slightly the dimensions of the existing plates and the biomechanical behavior of fixed mandible isn’t considered.

In order to decrease the need for plate removal, reduce the operative time and improve the stability of the fixation system, the biomechanical properties of intact mandible and treated mandible with a fracture are needed to investigated, and then a novel fixation plate could be designed based on biomechanical data using some optimal methods such as topological optimization. Topological optimization is one kind of structural optimization techniques that conducts the optimal design for a structure subject to presupposed loading and boundary conditions [14]. Optimal structure is acquired by reducing the material under no stress or less stress, after satisfying the stiffness requirements. And it is often achieved with combination of an optimization algorithm or a numerical method, such as finite element method [15]. In order to minimize the stress concentration of the structure, optimization based on density method that can reflect the essential characteristics of optimization is often used to pick out locations where material is necessary and join them to a macro-structure. Therefore, topological optimization is an effective tool to determine the shape of customized plate and location of the titanium screw.

In many vitro studies, cadaver mandibles or synthetic jawbone models were used to meet the measurable comparison of mechanical strength and stability of various fixation systems to cure mandibular angle fractures [16, 17]. But the disadvantages of using cadaver mandibles are that it is very difficult to obtain ideal samples and all of the mandibles differ from each other in quality. At the same time, synthetic mandibles lack the true comprehensive representation of biomechanical properties of vital jawbone [18]. FEM is widely used in obtaining detailed stress, strain and displacement distributions of the fractured mandible with fixation system. And it is verified by many studies that the results of the FEA is a valid, accurate, and non-invasive method to predict different parameters of the complex biomechanical behavior of human mandible [19–23].

The purpose of this study was to design a customized fixation plate for a mandibular angle fracture by topological optimization method based on a 3D model reconstructed from computerized tomography (CT) scan, which was verified by comparing to the conventional fixation systems on the biomechanical performance including stress, strain and displacement distributions of the fixed mandible system (including bone, plates and screws) calculated by FEA.

## Methods

**Fig. 1:**
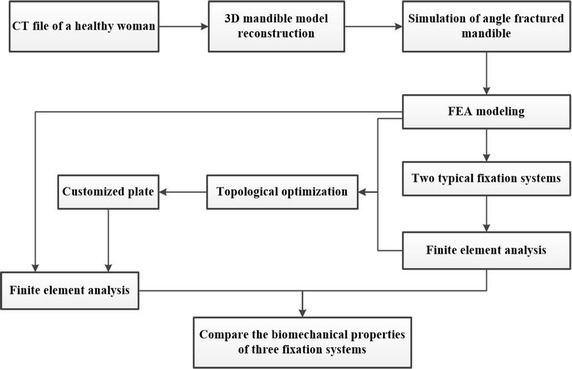
Flowchart of this study

The customized fixation plate was designed with topological optimization program in Abaqus (V6.14, Dassault Systèmes, Cedex, France) and fixed on a mandible with mimicked angle fracture in 3-matic (V9.0, Materialise, Leuven, Belgium, an accessory tool to MIMICS). A right side angle fracture with 1 mm gap was created on the 3D model in MIMICS (V16.0, Materialise, Leuven, Belgium). And then, two conventional fixation systems (type A—“one mini-plate”, type B—“two mini-plates”) were analyzed by FEM, and the result data were used as constraints for optimal design of customized plate, also taking into account the presence of the inferior alveolar nerve. After optimal design to get the customized plate, three angle fractured mandible models for three different fixation modalities were compared and analyzed: type A, type B, and type C-customized plate. The biomechanical performances of three internal rigid fixation systems to mandibular angle fracture with 1 mm fractured gap were investigated. Maximum von Mises stress, principal strain and displacement were measured from each numerical simulation. Totally, 27 data sets (3 loading positions × 3 fixation design × 3 outcome measurements) were compared and analyzed. Figure 1 shows the flowchart of this study, starting from the CT file of a healthy female, then comparing the results (stress, strain and displacement distribution) of the FEA, and finally validating the customized plate.

### 3D model reconstruction and fracture simulation

**Fig. 2:**
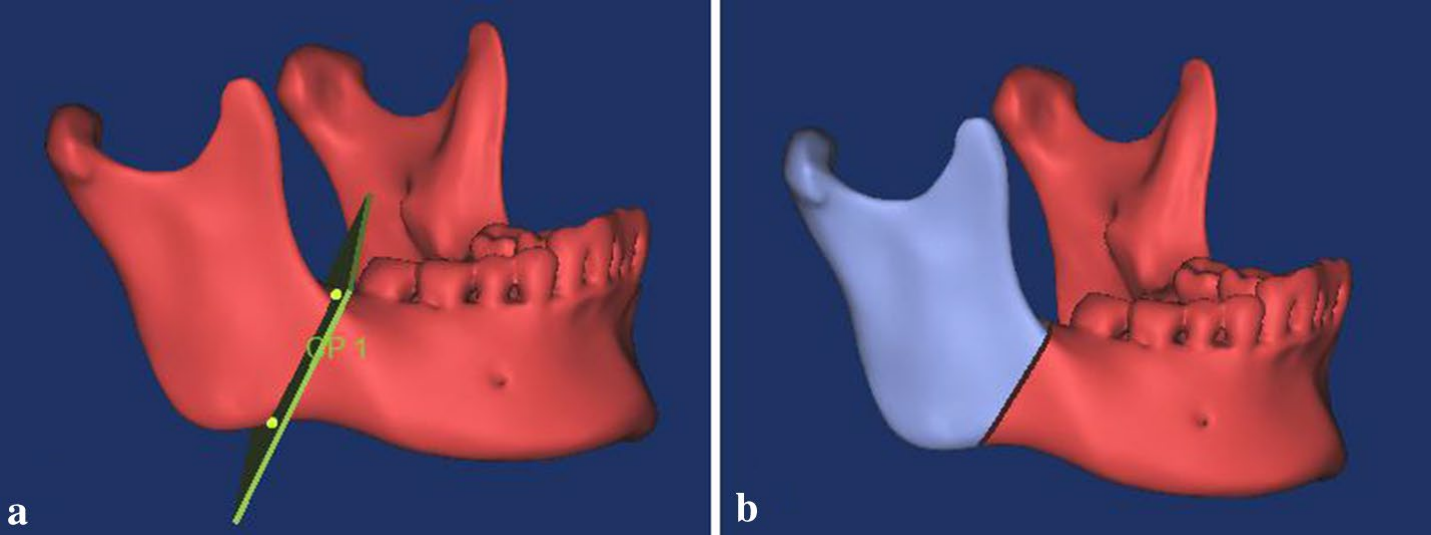
Simulation of the angle fractured mandible. **a** Intact mandible with 1 mm plane, **b** mandible with 1 mm fracture gap

A CT file of a healthy female was used to reconstruct a 3D model with contours of various hard tissues in MIMICS. Then, a 3D mandible model represented as triangular mesh was reconstructed as a new mask through the region of interest extraction. Finally, the angle fracture with 1 mm gap on the right side of the mandible was created by the cutting tool in the MIMICS (Fig. 2).

### FEA modeling and two typical fixation systems’ biomechanical analysis

Actually, the triangular meshes created from MIMICS just form a surface model. But volume meshes are required for finite element analysis. So the 3-matic was used to create high-quality volume meshes for the fractured mandible and fixation systems. The Abaqus was used to analyze the biomechanical performance of the fractured mandible with different internal rigid fixation modalities [18].

In many FEA studies, the material property of bone was assumed as a homogenous material or two kinds of materials including cortical and cancellous bone [9, 20, 24]. In reality, the mandible is inhomogeneous in material and consists of various bone components, so the bone locating at different areas have different modulus of elasticity and poisson’s ratio because of distinct degrees of calcification. Accordingly, some researchers reported that material property of bone could be calculated on Hounsfield unit (HU) value of CT images, and the real density could be related to modulus of elasticity. The material property of the mandible used in this paper was based on the following equations [25]:

1 $$\rho = 114 + 0.756 \times {\text{HU}}$$

2 $${\text{E}} = 0.51 \times \rho^{1.37}$$

**Fig. 3:**
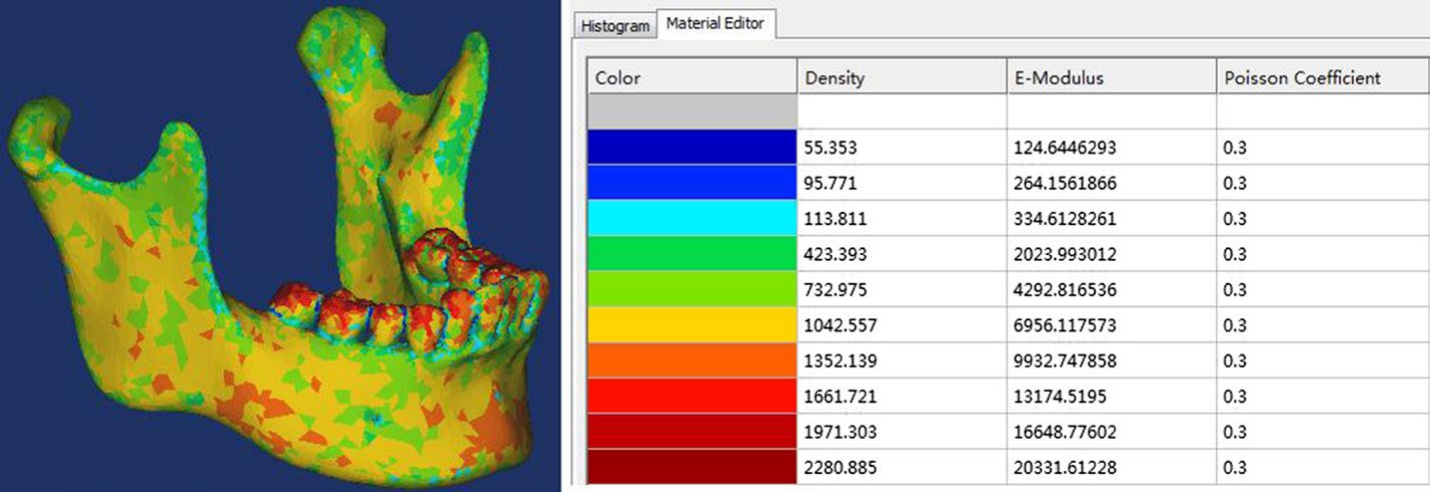
Material properties of the mandible model based on HU values from the CT images

The HU values of the mandible mask was evenly divided into ten groups in order to represent nouniform distribution of real mandibular properties (Fig. 3). The bone density ranged from 0.055 to 2.281 g/m^3^ based on CT file. The Young’s modulus ranged from 0.125 to 20.331 GPa based on the bone density. And the maximum Young’s modulus was distributed around the tooth, which was consistent with other studies [26]. The Young’s modulus was set as 116 GPa for titanium alloy (Ti-6Al-4V) plates and screws [27]. And all plates and screws have the same poisson’s ratio of 0.34.

**Fig. 4:**
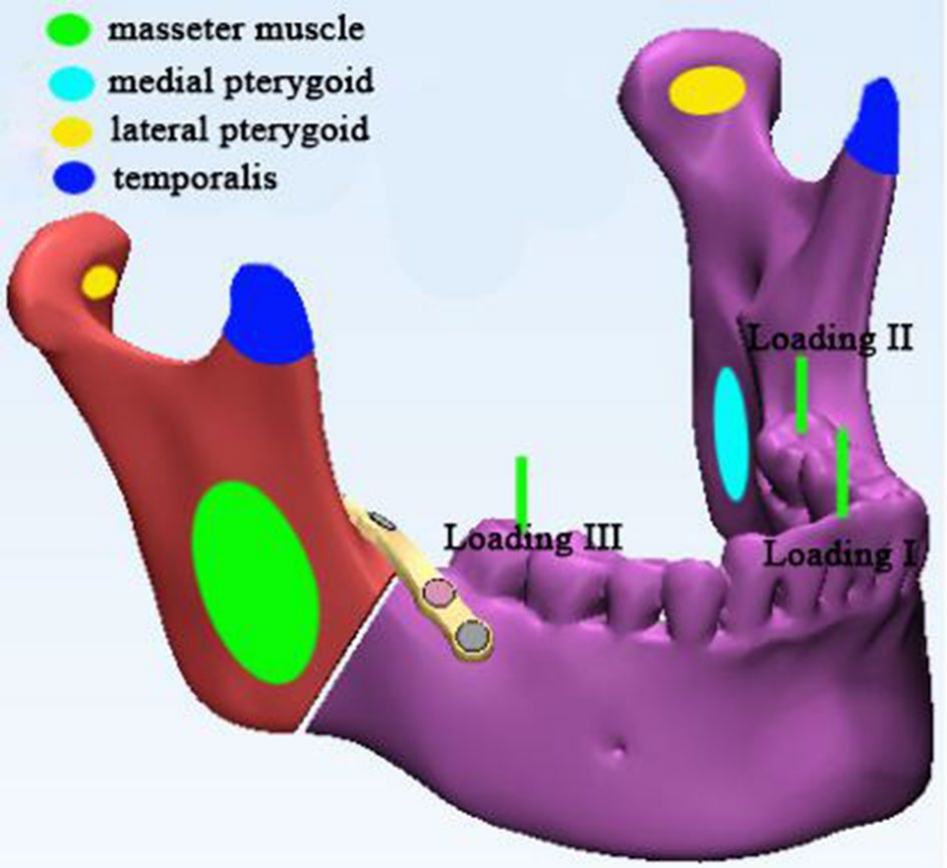
Loading and muscle areas on the mandible. Four kinds of mandible muscles were modeled as springs at the colored ovals, and the three green lines represented the three occlusal situations: loading I, incisor loading with 125 N, loading II, left second molar loading with 250 N, and loading III, right second molar loading with 250 N

Three occlusal situations simulated at different dental positions were applied to the angle fractured mandible model: loading I, incisor loading with 125 N, loading II, left second molar loading with 250 N, and loading III, right second molar loading with 250 N, and the directions of loads were all vertical (Fig. 4) [28]. Both condyles were fixed at 3 degrees of freedom, simulating the moment of biting. Mandible muscles were simulated as springs with no resistance during compression, the stiffness values of spring tension were taken from the related research [29]: masseter muscle = 16.35 N/mm, lateral pterygoid muscle = 12 N/mm, medial pterygoid muscle = 15 N/mm, and temporalis muscle = 14 N/mm, and the interaction area of the muscle were shown in Fig. 4. And the direction of vectors of the muscle structures were designed from published study [30]. The contact relation between bone sections along the fractured gap was set as “hard” contact. And the interaction of screw-bone, and screw-plate were set as “tie” constraint, without relative motions at these interfaces.

**Fig. 5:**
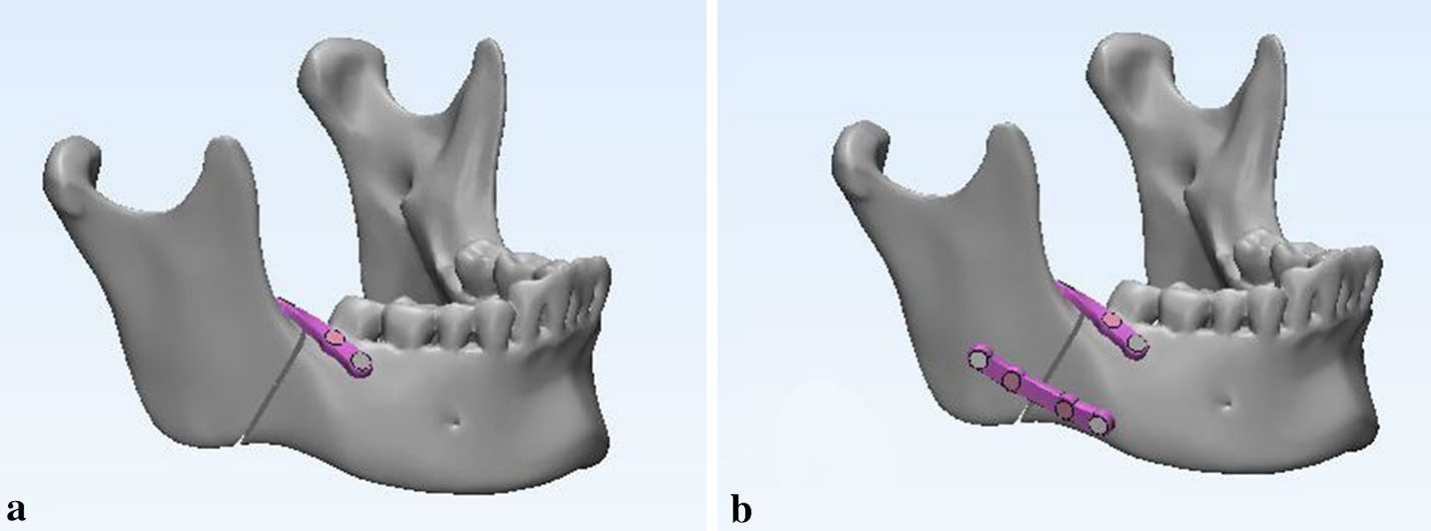
Two typical fixation systems for mandibular angle fracture. **a** Type A, one mini-plate fixed at the tension zone according to Champy’s technique, and **b** type B, upper mini-plate fixed at the same place of previous treatment, and a lower mini-plate fixed at the inferior border of the mandible

Two typical fixation systems including the plates and screws were modeled and fixed to the angle fractured mandible using 3-matic software: type A—“one mini-plate” (Fig. 5a); type B—“two mini-plates” (Fig. 5b). All screws were designed as cylinders with 2 mm diameter and 6 mm in length. All mini-plates with 2 mm thickness had four screw holes [6, 20, 31]. And the biomechanical properties of the fractured mandible fixed with the two different fixation systems were calculated by finite element analysis. As shown in Table 2, the maximum von Mises stress (MPa) of plate were 377, 332, and 394 for type A under the three loading conditions, and the values were 149, 123, and 192 for type B. As shown in Table 4, the maximum displacements (mm) of bony segments were 0.534, 0.484, and 0.635 for type A, and 0.352, 0.324, and 0.464 for type B. These data would be used as references and constraints for optimal design of customized plate.

### Customized fixation system optimal designing

**Fig. 6:**
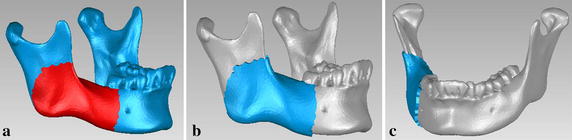
A original plate with 2 mm thickness. **a** Partial bone surface was selected, **b**, **c** different views of the generated plate

**Fig. 7:**
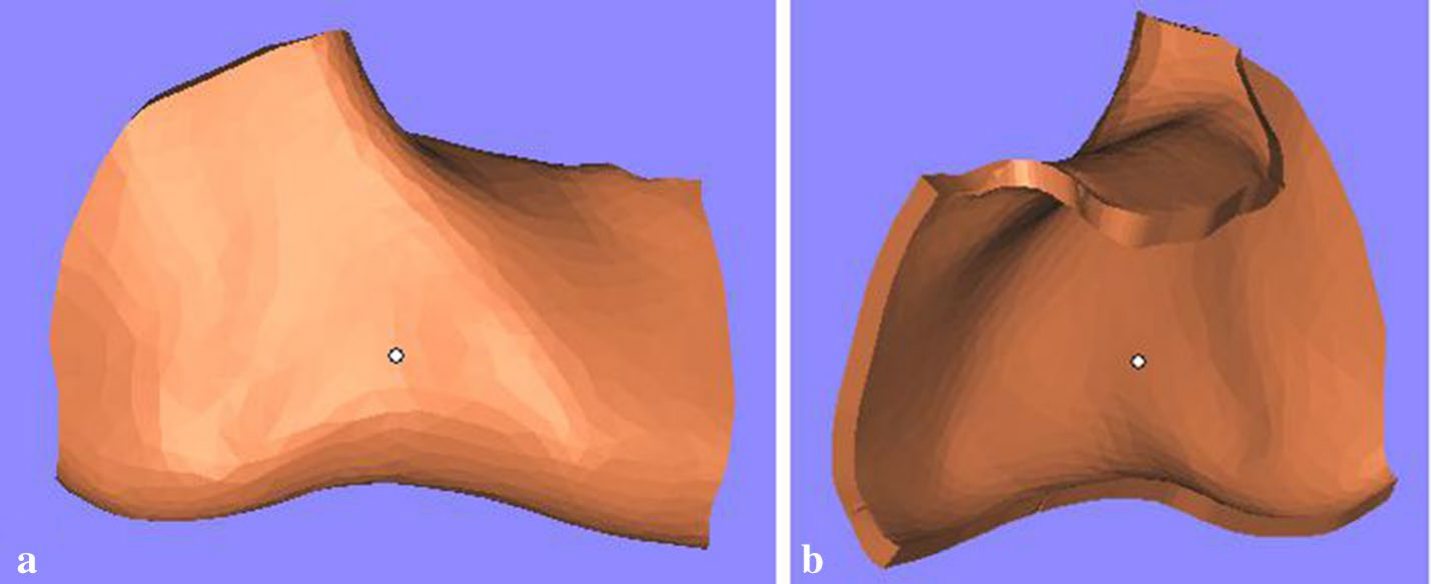
The edge of plate was cut and smoothed. **a** External surface of the modified plate, **b** internal surface of the modified plate

The customized fixation plate proposed in this paper is designed by the topological optimization program in Abaqus software. In order to find the valid scope of the customized plate, a 3D mandible model without right second molar was created by MIMICS software. Based on the mandible model with triangular mesh, an original plate with 2 mm thickness (Fig. 6) was created by extracting partial bone surface from the mandible in Geomagic Studio (V12.0, 3D system, Rock Hill, SC, USA). The thickness of 2 mm is designed from a study which concluded that 2 mm thick plate could show satisfactory result and adequate safety limits after comparing effects of plates with various thicknesses [3, 32]. Then, the original plate was transferred to Magics (V13.0, Materialise, Leuven, Belgium) to cut and smooth the edges of the plate (Fig. 7). Next, the smoothed plate and the mandibular angle fractured model were imported to Abaqus software to enter the process of topological optimization. There are two occlusal situations taken into account: incisor loading with 125 N and left second molar loading with 250 N, because the right second molar had been removed. And the other FEA modeling environment was same as the previous conditions, including muscles, condyles, and contact types. According to the FEA results of the two conventional fixation systems, the optimization design of the customized plate is represented in Table 1.

**Table 1:** Optimization design of the customized plate

Objective function	Minimize the volume of initial plate
Optimization constraints	The maximum stress of plate is under 120 MPa with loading I
	The maximum stress of plate is under 100 MPa with loading II
	The displacement of bony segment is under 0.3 mm with loading I
	The displacement of bony segment is under 0.3 mm with loading II

**Fig. 8:**
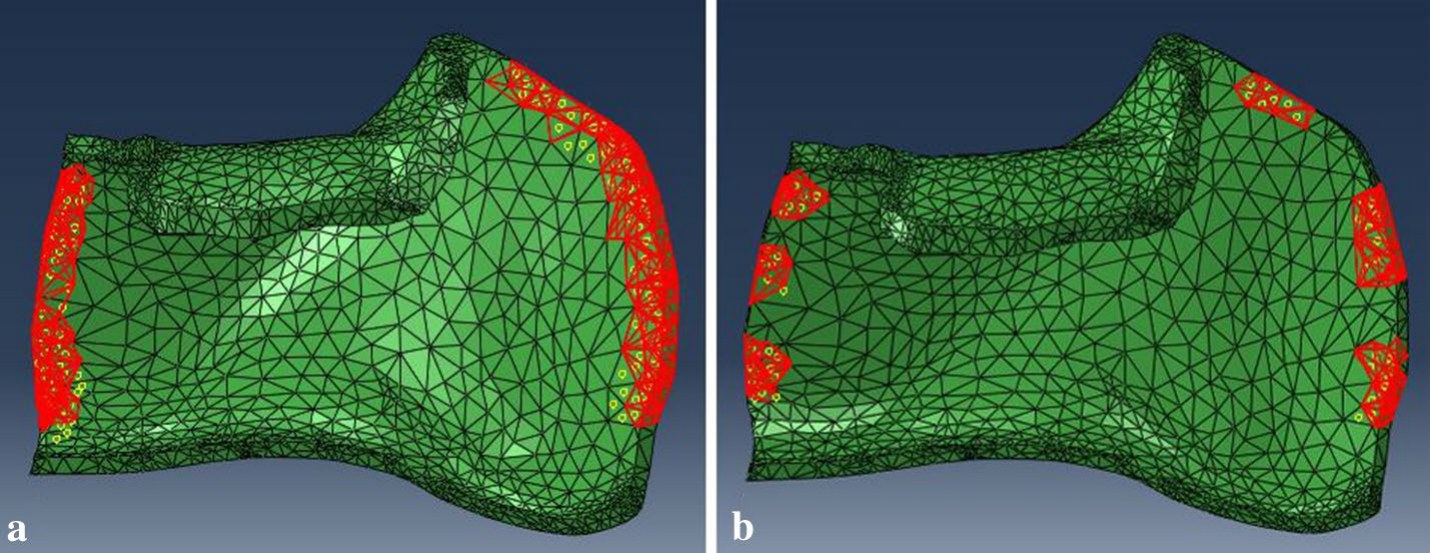
Two kinds of contact regions to mandible in topological optimization. **a** Strip tying, and **b** small segments tying

**Fig. 9:**
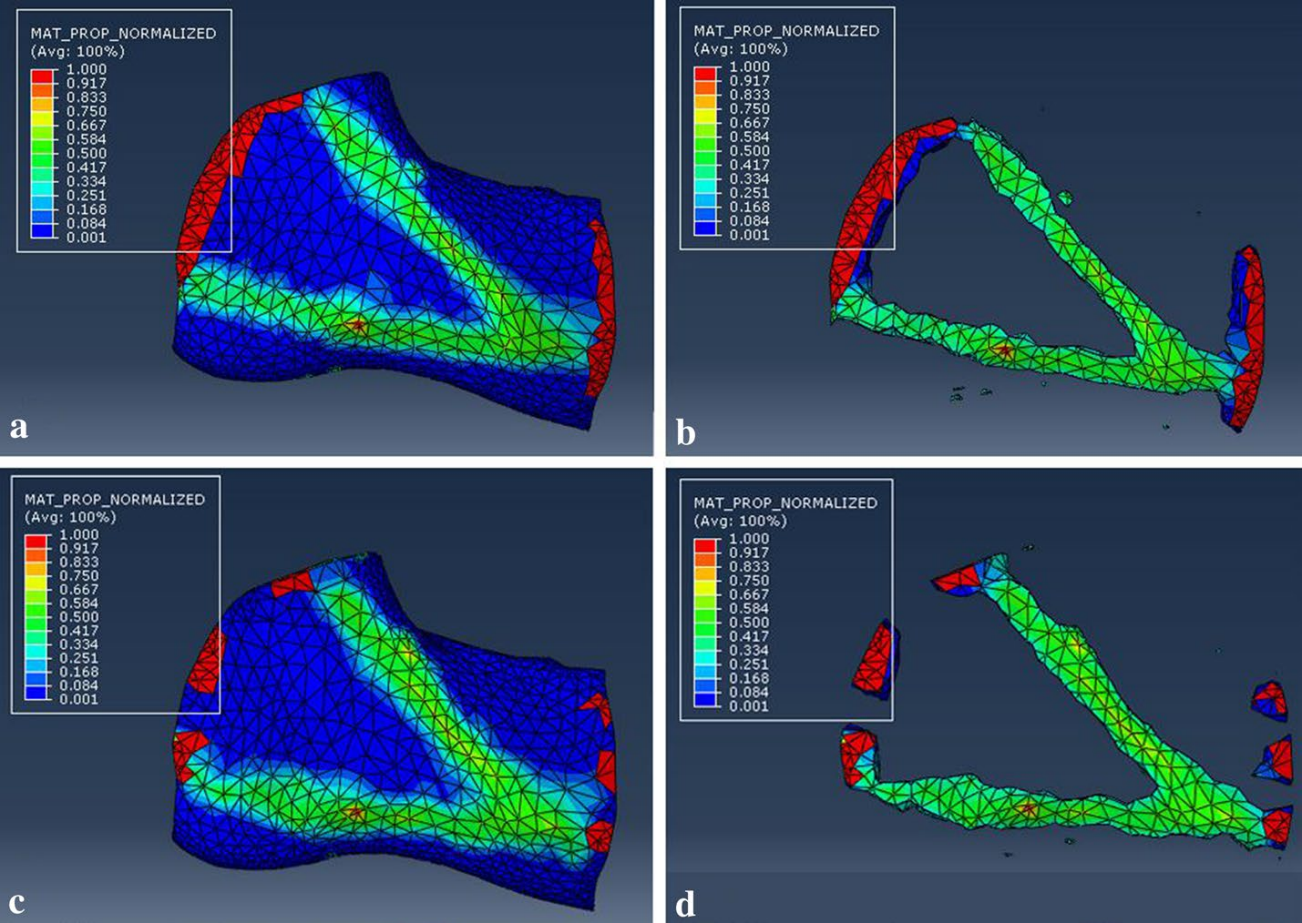
Results of the two topological optimization. Upper row: strip tying (**a** and **b**), lower row: small segment tying (**c** and **d**). The red regions are the frozen areas that will be reserved to the optimized results

**Fig. 10:**
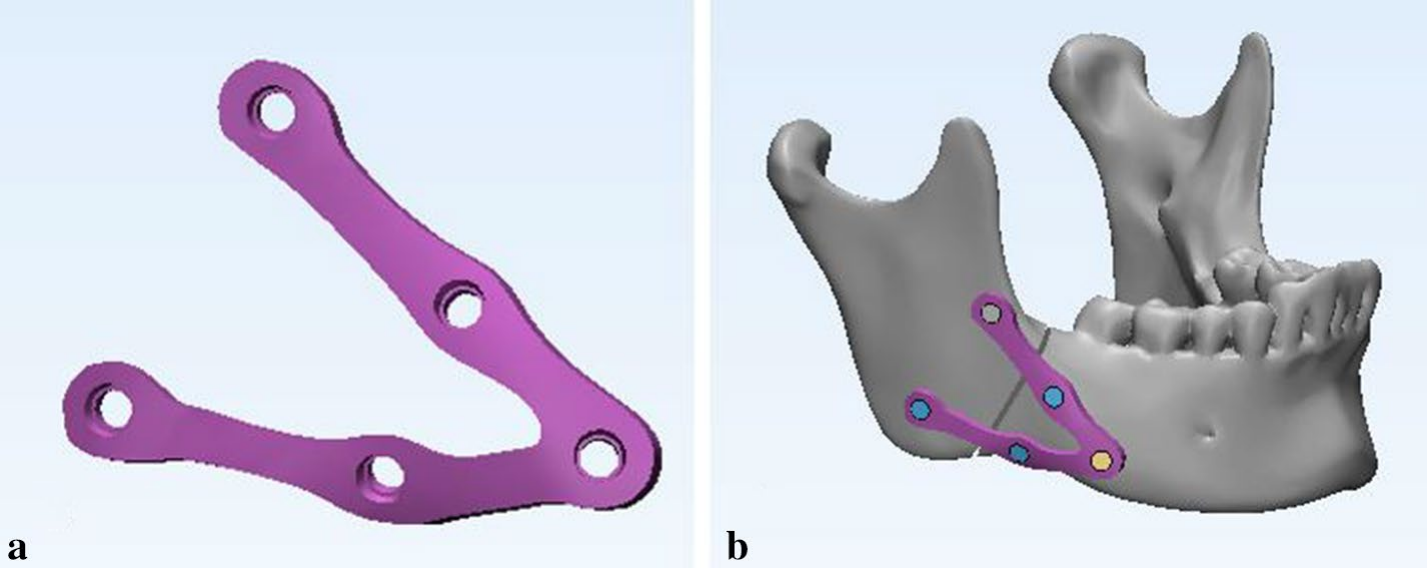
Fractured mandible with customized fixation system. **a** Final customized plate, and **b** customized fixation system (plate and screws) fixed on the fractured mandible

The mathematical model of the topological optimization based on the density method is shown as follow [11, 33, 34]:

3 $${\text{Find}}\;\;\;\;\;\;{\text{a}} = ( {\text{a}}_{ 1} , {\text{a}}_{ 2} ,\ldots , {\text{a}}_{\text{n}} )^{\text{T}}$$

4 $${\text{Min}}\;\;\;\;\;\;{\text{C(a)}} = {\text{F}}^{\text{T}} {\text{M}}$$

5 $${\text{S}} . {\text{t}} .\left\{ {\begin{array}{*{20}{lll}} {{{\rm{V}}^*} \le {\rm{V}}}\\ {{\rm{F}}\,{\rm{ = }}\,{\rm{NM}}}\\ {0<{{\rm{a}}_{\min }} \le {{\rm{a}}_{\rm{i}}} \le 1\quad\left( {{\rm{i = 1,2,}} \ldots {\rm{,n}}} \right)} \end{array}} \right.$$

a_i_ is design variable, the value is continuous between [a_min_, 1]; n is the number of optimum design variable; V is the volume of structure before optimization; V^*^ is the volume after optimization. F is the vector of structural force; N is the total stiffness matrix of the structure; M is the displacement vector of the structure. In addition, two groups of optimization were studied because of different contact regions between mandible and plate (Fig. 8), and the contact regions were froze during the process of optimization. Based on the results of the two types of topological optimization (Fig. 9), the customized plate was formed and completed in Magics software. Finally, design the screws for the customized plate in the 3-matic software after fixing and smoothing it in the Magics software (Fig. 10). To simplify the modeling process, the screws were all designed as cylinders, and were 6 mm in length for customized plate.

### Comparisons for biomechanical behaviors of three fixation systems by FEA

The biomechanical properties of angle fractured mandible with customized fixation design was calculated by finite element analysis under the same FEA modeling conditions (same angle fractured mandible model and same loading conditions) of the two typical fixation systems. And the maximum von Mises stress, strain and displacement of fixed mandible system including bone, plate and screws were collected and analyzed in Tables 2, 3 and 4.

**Table 2:** Maximum von Mises stress (MPa) of three different fixation modalities

	Loading I	Loading II	Loading III
Bone			
Type A	66	80	103
Type B	57	65	56
Type C	48	44	63
Fixation system (plate and screw)			
Type A	377	332	394
Type B	149	123	192
Type C	97	81	116

**Table 3:** Maximum principal strain (%) of three different fixation modalities

	Loading I	Loading II	Loading III
Bone			
Type A	0.007	0.009	0.009
Type B	0.009	0.013	0.008
Type C	0.007	0.006	0.009
Fixation system (plate and screw)			
Type A	0.003	0.003	0.003
Type B	0.001	0.001	0.002
Type C	0.001	0.001	0.001

**Table 4:** Maximum displacement (mm) of bony segment along the fractured gap

	Loading I	Loading II	Loading III
Type A	0.534	0.484	0.635
Type B	0.352	0.324	0.464
Type C	0.286	0.180	0.246

## Results

**Fig. 11:**
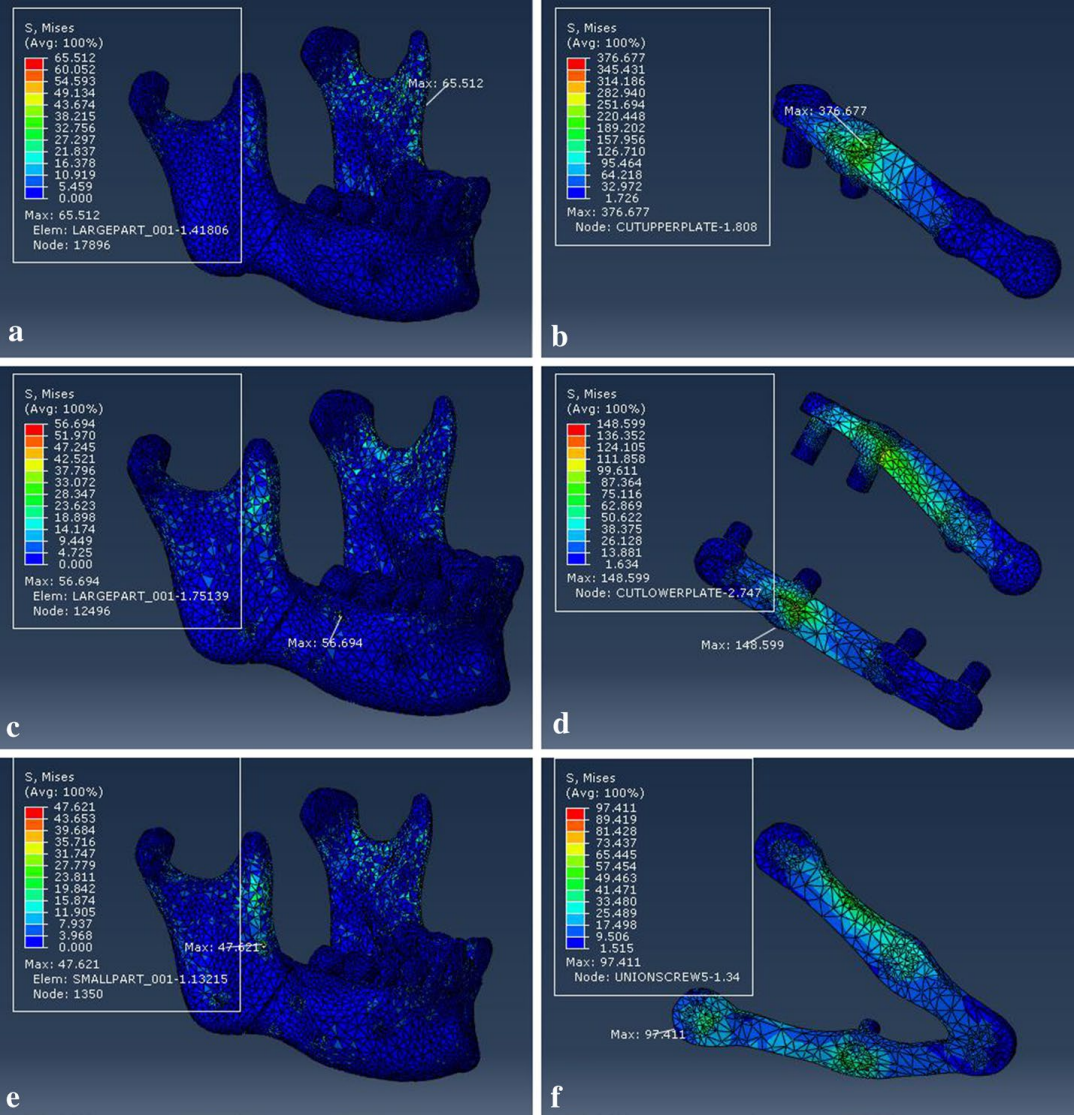
von Mises stress distribution to the mandible and fixation system under loading I. Type A (**a** and **b**), type B (**c** and **d**), and type C (**e** and **f**)

**Fig. 12:**
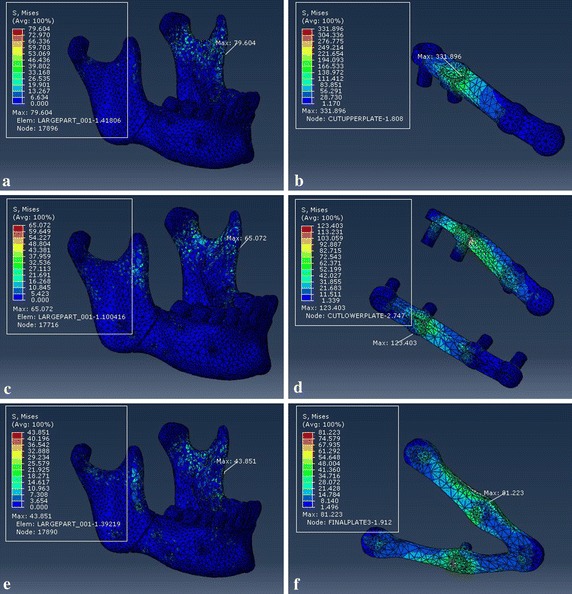
von Mises stress distribution to the mandible and fixation system under loading II. Type A (**a** and **b**), type B (**c** and **d**), and type C (**e** and **f**)

**Fig. 13:**
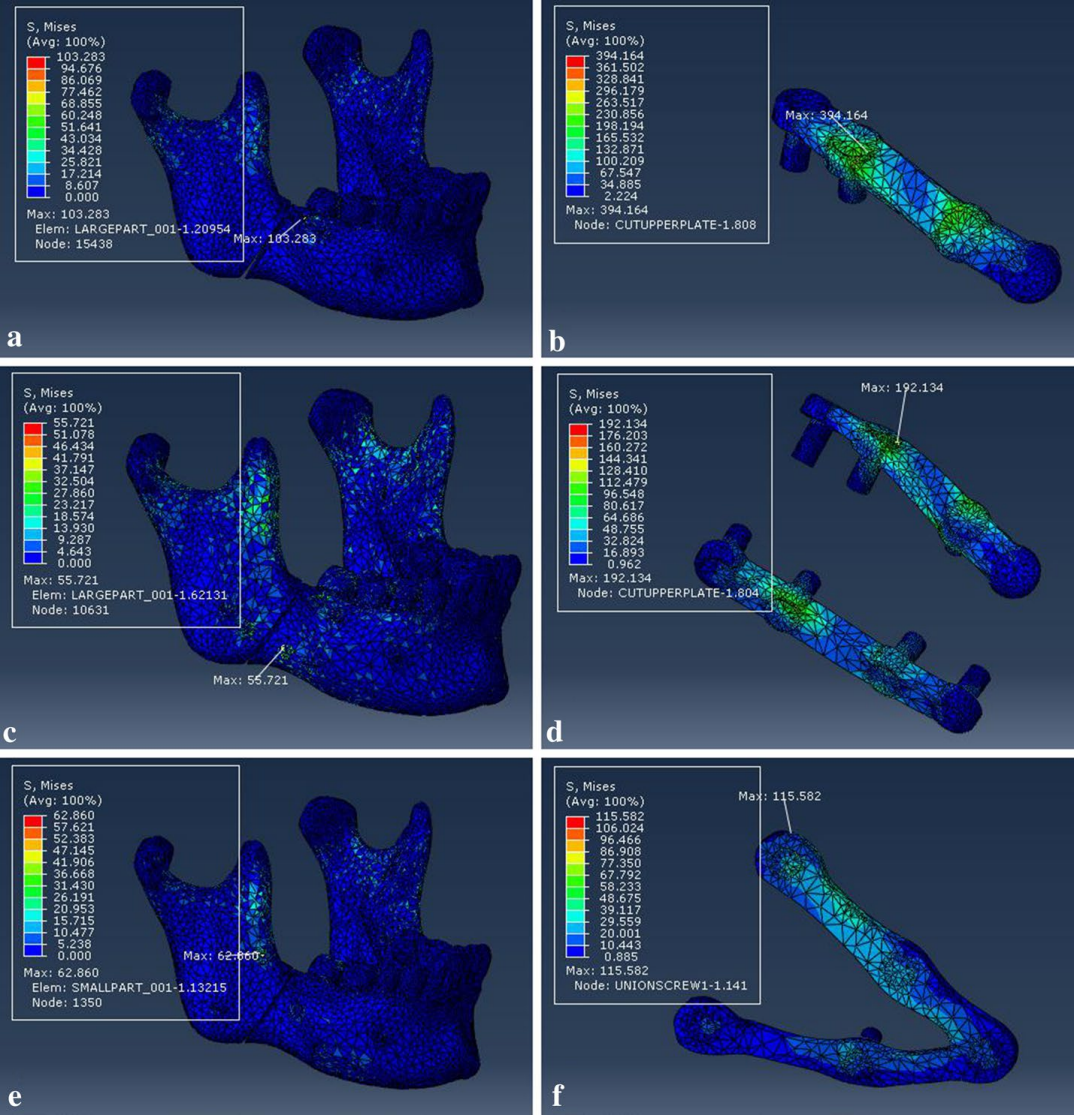
von Mises stress distribution to the mandible and fixation system under loading III. Type A (**a** and **b**), type B (**c** and **d**), and type C (**e** and **f**)

In order to get the detailed information of the effects of the customized fixation designs, the FEA results of customized plate and the other two conventional fixation systems are shown in Figs. 11, 12 and 13, which represented respectively the stress distribution of the angle fractured mandible and fixation system (plates and screws) under three occlusal situations. In each figure, the left column displays the stress distribution of mandible, and right column displays the stress distribution of the fixation system. And the maximum stress emerged at the mandibular ramus or screw holes regardless of the fixation designs.

**Fig. 14:**
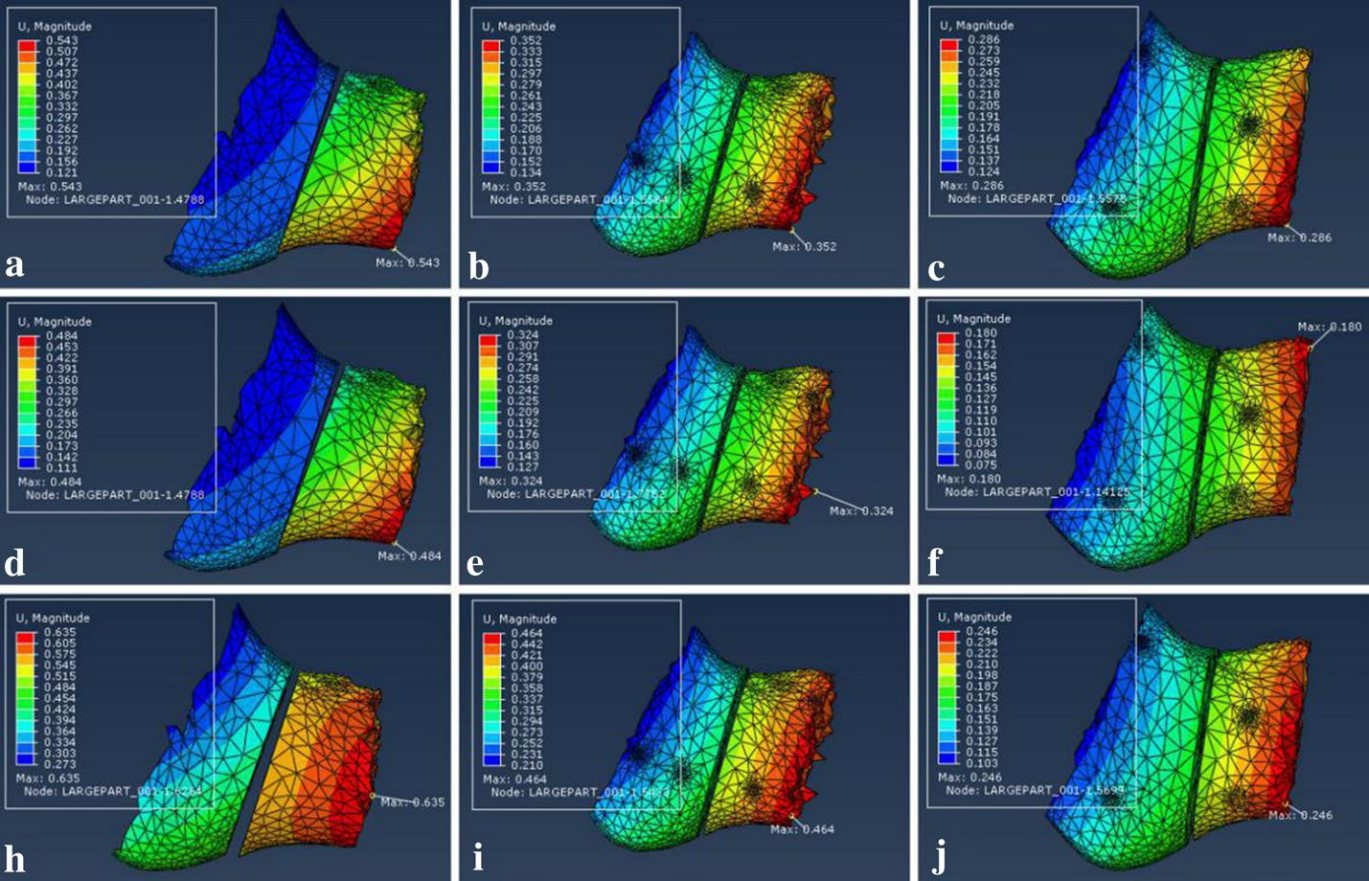
Displacement of bony segment along the fracture gap under three different loading locations. Upper row: loading I—**a** Type A, **b** type B and **c** type C. Middle row: loading II—**d** type A, **e** type B and **f** type C. Lower row: loading III—**h** type A, **i** type B and **j** type C

Figure 14 shows the displacement of bony segments along the 1 mm fracture gap with three different fixation systems. And the maximum displacement was located at the area near the chin regardless of the loading positions.

Tables 2 and 3 record the results of maximum von Mises stress and maximum principal strain of the separated fractured mandible and fixation system under the same loading locations. For loading I, the maximum stress to fixation system were 377, 149, and 97 MPa for type A, type B, and type C, respectively. And the values of type C fixation were lower than other two fixation systems regardless of loading positions.

Table 4 shows the maximum displacement of bony segments along the 1 mm fracture gap under three loading locations. And the maximum displacement under loading II were 0.484, 0.324, and 0.180 mm for fixation type A, type B, and type C, and the values under loading II were lower than other two loading conditions.

## Discussion

The topological optimization method can be conducted based on finite element analysis to design the most appropriate plate structure for fractured mandible using the minimum output values for stress of plate and displacement of bony segments to seek maximum reduction volume on an original plate. As shown in Table 2, under the same three loading conditions, the maximum von Mises stress (MPa) of plate were 377, 332, and 394 for type A, and 149, 123, and 192 for type B. The values of customized fixation system decreased 74.3, 75.6 and 70.6% when compared to type A, and 34.9, 34.1, and 39.6% compared to type B, respectively. As shown in Table 4, the maximum displacement (mm) of bony segments were 0.534, 0.484, and 0.635 for type A, and 0.352, 0.324, and 0.464 for type B. And the values of customized fixation system decreased 46.4, 62.8 and 61.3% to type A, and 18.8, 44.4, and 47.0% to type B.

According to literature, the mini-plate is widely used in all types of mandibular fractures, but clinical work found that the mini-plate may be insufficient in strength and stability, which will lead to failure of treatment to fractured mandible. Consequently, designing a customized fixation system that satisfy the stress condition and produce high stability is very essential. Topology optimization can be considered as an efficient way to achieve objective of reducing stress concentration [34]. It has been used to design micro-structure that enhances formation requirement with different pore sizes, hierarchical scaffold [15], however, it is seldom used to design structure to angle fractured mandible. The design method of topological optimization in this paper was based on the “smaller the better” quality characteristics for the reduction of plate volume and the improvement of stability of mandible segments (small bone plate stress and relative movement) [13, 15]. The optimized results shown in Fig. 9 indicate that different contact areas have no obvious influence on the topological optimization, so the final customized plate is designed as the “V” pattern plate shown in Fig. 10a. Above all, the design methodology for customized fixation system could be used for other fractures in mandible or other bones to acquire better mechanical behavior of the system and improve stable environment for bone healing.

The mandibular muscles were modeled as springs to simulate real environment of human mandible, which can effectively analyze the mechanical performance of the fractured mandible with different fixation modalities. In addition, the material properties of mandible in this paper were assigned by the HU values of CT images, which will be more appropriate than the method that just takes use of two material (cortical and cancellous bone) of mandible [28], because the human mandible is an inhomogeneous material components. In addition, our FEA results were studied under incisor and second molar loading positions that are considered as the most common occlusal situations.

Finite element method is an effective way to accurately predict the stress and strain distribution of the mandible and plate after surgery, which can avoid the phenomenon that the plate or screw was damaged for concentrated force after fixation. Compared with conventional experiments, FEM has the advantages of reducing complexity of experimental operation and error of manual operation. At the same time, FEM can keep the consistency of the mandible models, effectively reducing the error from comparing the biomechanical properties of different fixation methods, and it is beneficial to choose a appropriate way to treat angle fractured mandible, reducing the failure of operation.

The corresponding maximum von Mises stresses and principal strains for the fixed mandible system were predicted in Tables 2 and 3. The FEA results represent that the maximum stresses of all three fixation systems are under the yield strength of titanium alloy (σ = 780–950 MPa) [35], so all of the fixation systems are safe for fracture treatment. But the stress distribution of customized plate is more uniform than conventional fixation mini-plate regardless of loading locations. And the maximum stress of mandible that located at the screw hole or mandibular ramus is well below the allowable stress of human bone ([σ] = 140 MPa) [36, 37], which will avoid bone resorption and achieve the stability of fixation plate. Critical yield tensile strain of human cortical bone is 0.4% according to relevant studies [18, 38]. And all principal strains of fixed mandible systems were well below it regardless of occlusal situations. The displacement distribution of bony segments were shown in Fig. 14, and the maximum displacement were collected in Table 4. According to involved studies, when a minimal fracture gap is under 150 μm and the fixation environment is stable, it is possible to achieve primary healing; when a small gap is under 2 mm with a stable mechanical environment, it is able to fulfill a good secondary healing [39]. So all of three fixation systems are beneficial to bone healing, but the customized fixation design has better biomechanical performance of treating fractured mandible.

The customized fixation system designed directly from the specific patient is much fitter to the bone surface when compared with the standard mini-plate, which will reduce the time of bending and fixing the plate during operation. Currently, type B is the most popular treatment in clinical cases to mandibular angle fracture, so it is persuasive to compare the FEA results of the customized fixation system with it. Firstly, the customized fixation system just have five screws, which will be less damaging to mandible and will reduce complication of infection [40], while the type B needs eight screws to fix the fractured gap. Secondly, the customized plate is an integrated plate with two branches, which will enhance stability of fixation under the different loads and make accurate positioning for surgery. Thirdly, the distances between screw holes in customized plate are larger than that in standard mini-plate, and the distances between screw holes can be changed for different patients, especially for patients with bad bone condition.

The customized plate can be fabricated by selective laser melting (SLM) rapidly to satisfy the urgent requirements for surgical treatment. SLM, a typical technique of additive manufacturing, could produce titanium plate from metal powder, which has been verified in clinical application [41, 42]. Therefore, combined with SLM, topological optimization could allow a customized plate for mandible fracture fixation be designed and fabricated rapidly and used in clinic to improve the surgery quality and efficiency.

## Conclusions

The customized fixation system with topological optimized structure has good biomechanical behavior for mandibular angle fracture because the stress, strain and displacement within the plate could be reduced significantly comparing to conventional “one mini-plate” or “two mini-plates” systems. The design methodology for customized fixation system could be used for other fractures in mandible or other bones to acquire better mechanical behavior of the system and improve stable environment for bone healing. And together with SLM, the customized plate with optimal structure could be designed and fabricated rapidly to satisfy the urgent time requirements for treatment.

## Abbreviations

FEA: finite element analysis
FEM: finite element method
3D: a three-dimensional
CT: computerized tomography
HU: Hounsfield unit
SLM: selective laser melting
